# Lenalidomide plus R-CHOP21 in newly diagnosed diffuse large B-cell lymphoma (DLBCL): long-term follow-up results from a combined analysis from two phase 2 trials

**DOI:** 10.1038/s41408-018-0145-9

**Published:** 2018-11-08

**Authors:** A. Castellino, A. Chiappella, B. R. LaPlant, L. D. Pederson, G. Gaidano, W. R. Macon, G. Inghirami, C. B. Reeder, A. Tucci, R. L. King, A. Congiu, J. M. Foran, V. Pavone, C. E. Rivera, M. Spina, S. M. Ansell, F. Cavallo, A. L. Molinari, Giovannino Ciccone, T. M. Habermann, T. E. Witzig, U. Vitolo, G. S. Nowakowski

**Affiliations:** 1 Azienda Ospedaliero Universitaria Città della Salute e della Scienza di Torino, Torino, Italy; 2 Mayo Clinic, Rochester, MN USA; 3 Department of translational Medicine, University of Eastern Piedmont, Novara, Italy; 4 Cornell University, New York, NY USA; 5 Mayo Clinic, Scottsdale, AZ USA; 6 Azienda Ospedaliera Spedali Civili di Brescia, Brescia, Italy; 7 IRCCS Azienda Ospedaliera Universitaria San Martino - IST Istituto Nazionale per la Ricerca sul Cancro, Genova, Italy; 8 Mayo Clinic, Jacksonville, FL USA; 9 Unit of Hematology and Hemopoietic Stem Cell Transplantation, Ospedale Cardinale G Panico, Tricase, Italy; 10 Division of Medical Oncology, Centro di Riferimento Oncologico Aviano National Cancer Institute, Aviano, Italy; 11 Department of Oncology and Hematology, Infermi Hospital, Rimini, Italy; 12 Unit of Clinical Epidemiology, CPO, Azienda Ospedaliero Universitaria Città della Salute e della Scienza di Torino, Torino, Italy

## Abstract

Lenalidomide-RCHOP (R2-CHOP21) has been shown to be safe and effective in patients with untreated diffuse large B-cell lymphoma (DLBCL). The aim of this analysis is to report long-term outcome and toxicities in newly diagnosed DLBCL patients who received R2-CHOP21 in two independent phase 2 trials, conducted by Mayo Clinic (MC) and Fondazione Italiana Linfomi (FIL). All patients received R-CHOP21 plus lenalidomide. Long-term progression-free survival (PFS), time to progression (TTP), overall survival (OS) and late toxicities and second tumors were analyzed. Hundred and twelve patients (63 MC, 49 FIL) were included. Median age was 69 years, 88% were stage III–IV. At a median follow-up of 5.1 years, 5y-PFS was 63.5%, 5y-TTP 70.1% and 5y-OS 75.4%; according to cell of origin (COO): 5y-PFS 52.8% vs 64.5%, 5y-TTP 61.6% vs 69.6% and 5y-OS 68.6% vs 74.1% in germinal center (GCB) vs non-GCB respectively. Four patients experienced grade 4–5 late toxicities. Grade ≤ 3 toxicities were infections (*N* = 4), thrombosis (*N* = 1) and neuropathy (*N* = 3). Seven seconds tumors were observed. Long-term follow-up demonstrates that R2-CHOP21 efficacy was maintained with high rates of PFS, TTP, and OS. Lenalidomide appears to mitigate the negative prognosis of non-GCB phenotype. Incidence of therapy-related secondary malignancies and late toxicities were low.

## Introduction

The addition of the anti-CD20 monoclonal antibody rituximab, to a chemotherapy regimen with cyclophosphamide, doxorubicin, vincristine, and prednisone (R-CHOP) dramatically improved the outcome of patients affected by diffuse large . B-cell lymphoma (DLBCL) and became the standard of treatment^1–4^. However, about 40% of patients relapse or do not respond to initial chemoimmunotherapy, and patients with relapsed DLBCL have a poor prognosis^5,6^

Attempts to improve the efficacy of front-line therapy have included dose-dense and dose-intensified regimens followed by autologous stem cell transplantation (ASCT)^7–10^, the use of different induction schedules^11^, early intensification of rituximab administration^12^, or the substitution of obinutuzumab for rituximab^13^. However they did not improve the outcome of DLBCL patients^14^.

The biological complexity of DLBCL suggests that a tailored therapeutic approach based on the biological and molecular signature might be a promising strategy. Gene-expression profiling (GEP) identified two major DLBCL subtypes: the germinal center B-cell like (GCB) and the activated B-cell like (ABC) also refered to as GCB and non-GCB in immunohistochemistry (IHC)-based subtyping^15,16^. DLBCLs with ABC (non-GCB) phenotype are associated with an unfavorable outcome when treated with standard R-CHOP ^17^.

Lenalidomide is an immunomodulatory drug (ImiD) that demonstrates significant activity in relapsed/refractory lymphomas in monotherapy and in combination with rituximab^18,19^. In xenograft models of DLBCL, lenalidomide demonstrated major clinical activity on ABC-subtype, in which there was downregulation of B-cell receptor-dependent NF-kB through an inhibition of the transcription factor interferon regulatory factor 4 (IRF4) and cerebron^20^. Lenalidomide was subsequently investigated as front-line treatment of DLBCL in combination with standard R-CHOP chemoimmunotherapy administered every 21 days (R2-CHOP21). Two phase I/II studies, conducted by Mayo Clinic (MC078E trial)^21,22^ and by Fondazione Italiana Linfomi (FIL, REAL07 trial)^23,24^ reported that the combination regimen R2-CHOP21 is feasible and effective, with an overall response rate (ORR) greater than 90%. These early results have led to two currently ongoing randomized trials^25,26^. However, long-term efficacy and safety of this regimen are not well defined. Here, we report the results of a combined analysis on a long-term follow-up of the efficacy and safety in newly diagnosed DLBCL patients who received R2-CHOP21 in these two independent single-arm phase 2 studies.

## Methods

### Study design and participants

This analysis included all patients with newly diagnosed histologically-confirmed CD20+ DLBCL that were enrolled in two R2-CHOP21 phase 2 trials; one conducted by Mayo Clinic (MC078E trial^22^) between September 2008 and August 2013, and one conducted by Fondazione Italiana Linfomi (REAL07 trial^24^) between April 2010 and June 2011.

MC078E was an investigator-initiated, open-label, single-arm phase I/II study, while REAL07 was an open-label, multicentre phase I/II trial that was conducted in 13 centers in Italy and one center in Germany.

Patients in phase II and those from phase I that were administered the maximum tolerated dose of lenalidomide were included in this present long-term analysis.

The main differences between the two populations were that the MC078E trial included all patients older than 18 years without an upper age limit and all International Prognostic Index (IPI) risk scores, while the REAL07 trial focused on patients aged between 60 and 80 years old and included only patients with IPI score ≥ 2, excluding low risk IPI score cases. Other inclusion criteria for both studies were similar and included the following: Ann Arbor Stage II–IV; measurable disease with at least one lesion ≥ 1.5 cm in a single diameter by CT; Eastern Cooperative Oncology Group (ECOG) PS 0–2; estimated cardiac ejection fraction ≥ 45% (MUGA scan or echocardiogram); and preserved organ functions^22,24^. Main exclusion criteria included the following: presence of central nervous system (CNS) involvement at diagnosis, HIV, HCV, and HBV infection, history of life-threatening or recurrent thrombosis and/or embolism, unless they were receiving anticoagulant therapy during the protocol treatment; history of any neoplasia in the previous 3 years.

### Procedures

All patients included in the analysis received standard R-CHOP21 treatment in association with lenalidomide administered in different schemes in the MC078E and REAL07 trials.

Both studies utilized standard R-CHOP21 therapy (rituximab 375 mg/m^2^, cyclophosphamide 750 mg/m^2^, doxorubicin 50 mg/m^2^, vincristine 1.4 mg/m^2^ (capped at 2 mg), all on day 1, prednisone 100 mg/m^2^ (MC078E) or 40 mg/m^2^ (FIL-REAL07) per day on days 1 through 5, given every 21 days).

In the MC078E trial, lenalidomide was administered at a dose of 25 mg/day for 10 days/cycle (MTD determined in the phase I trial^21^), whereas in the REAL07 study, lenalidomide dose was 15 mg/day for 14 days/cycle (MTD determined by phase 1 stydy^23^). Accordingly, the total cumulative doses of lenalidomide in both studies were similar: 250 mg/cycle and 210 mg/cycle in Mayo Clinic and FIL study respectively. CNS prophylaxis with 12 mg of intrathecal methotrexate (IT MTX) was administered in patients with a high risk of CNS progression/relapse according to local clinical practice. All patients received primary prophylaxis for neutropenia, with a 6 mg pegfilgrastim subcutaneous injection on day 2 in MC078E trial and with granulocyte colony-stimulating factors in REAL07 trial. Prophylaxis for deep vein thrombosis was as follows in both trials: low dose aspirin (acetylsalicylic acid), 81 mg per day and prophylactic low molecular weight heparins in MC078E and REAL07 trials respectively. Pneumocystis Jirovecii infection prophylaxis with co-trimoxazole or pentamidine aerosol was administered according to local clinical practice. Occult carriers of hepatitis B virus were treated with lamivudine. A pre-phase treatment with steroids or vincristine in cases of urgent clinical need was allowed in 7 days before study treatment. Tumor lysis prophylaxis, antiemetics, and supportive care were the standard of care and at the discretion of the treating physician.

### Pathology review and DLBCL profile assessment

All histologic diagnoses were confirmed by a central pathology review using WHO 2008 classification diagnostic criteria^27^ in both trials. COO subtype was determined by Hans algorithm IHC as either GCB or non-GCB ^15^.

In MC078E trial, the histological diagnoses and COO phenotype were validated independently at the British Columbia Cancer Agency on a subset of the study cases (*N* = 43) for standardization. There was agreement with the assessment of GCB versus non-GCB in 93% (40 of 43) of cases. As for the REAL07 trial, two pathologists centrally reviewed diagnostic lymphoma samples from each patient for IHC COO. Differences of opinion were resolved by joint review with a multihead microscope.

### Outcome

Progression-free survival (PFS) was defined as the time from the date of registration until the date of disease progression or death due to any cause. Time to progression (TTP) was defined as the time from the date of registration until the date of disease progression. Overall survival (OS) was defined as the time from the date of registration until the date of death as a result of any cause. Additional analyses of the association between outcome and cell of origin and IPI were performed.

The incidence of CNS relapses, late toxicities, and secondary malignancies was reported.

Late toxicities were defined as any type of toxicity that was reported from the date of the treatment completion until the date of the last follow-up and recorded as being possibly, probably or definitely related to study treatment. Adverse events were defined as per National Cancer Institute Common Terminology Criteria for Adverse Events v 4.03. Toxicities were described as maximum grade occurred for each patient.

### Statistical methods

The MC078E trial was a phase II study that utilized a one-stage binomial design to assess the efficacy and tolerability of R2-CHOP21 regimen with 93% power and 9% type I error rate. The REAL07 trial was a phase II study designed according to Simon’s two stage minimax design, with 80% power and 5% type I error rate.

In the present long-term combined analysis from the two trials, all patients were analyzed as a single cohort. Long-term outcome results from the two trials separately were also reported. The distribution of time-to-event survival end points were estimated by using Kaplan Meier methods. Differences between groups were evaluated by log-rank statistics. For PFS and TTP, patients were censored on the date of their last disease assessment. For OS, patients were censored on the date of last follow-up. A subgroup analysis of PFS, TTP, and OS by COO phenotype and IPI was performed.

### Ethics

The trial was done in accordance with the Declaration of Helsinki and good clinical practice guidelines. Approval was obtained from the independent ethics committees and institutional review boards at each site before trial initiation. All patients provided written informed consent to participate in the study.

## Results

Between September 2008 and August 2013, 63 patients with DLBCL were enrolled in the MC078E trial and were included in the present long-term follow-up analysis. In the REAL07 phase II trial, 49 patients, enrolled between October 2008 and June 2011, were included. Hence, the entire cohort included 112 patients (63 MC078E, 49 REAL07) with de-novo DLBCL.

### Clinical characteristics

The median age of the whole cohort was 69 years (range 22–87 years), with 50 (44.6%) patients over age 70 years and five (4.5%) patients over age 80. Main clinical characteristics are summarized in Table 1.

**Table 1:** Patient characteristics

	MC078E (*N* = 63)	REAL07 (*N* = 49)	Total (*N* = 112)
Age at diagnosis			
*N*	63	49	112
Mean (SD)	64.5 (13.24)	69.4 (5.16)	66.7 (10.74)
Median	67.0	69.0	69.0
Range	22.0, 87.0	61.0, 79.0	22.0, 87.0
Sex, *n* (%)			
Male	39 (61.9%)	29 (59.2%)	68 (60.7%)
Female	24 (38.1%)	20 (40.8%)	44 (39.3%)
Stage, *n* (%)			
I/II	8 (12.7%)	6 (12.2%)	14 (12.5%)
III/IV	55 (87.3%)	43 (87.8%)	98 (87.5%)
Systemic symptoms, *n* (%)			
A	30 (65.2%)	26 (53.1%)	56 (58.9%)
B	16 (34.8%)	23 (46.9%)	39 (41.1%)
Missing	17	0	17
CNS-IPI, *n* (%)			
1	6 (9.5%)	1 (2.0%)	7 (6.3%)
2	22 (34.9%)	19 (38.8%)	41 (36.6%)
3	25 (39.7%)	16 (32.7%)	41 (36.6%)
4	6 (9.5%)	10 (20.4%)	16 (14.3%)
5	4 (6.3%)	3 (6.1%)	7 (6.3%)
IPI Group, *n* (%)			
0–2	29 (46.0%)	20 (40.8%)	49 (43.8%)
3+	34 (54.0%)	29 (59.2%)	63 (56.3%)
COO, *n* (%)			
GCB	33 (55.9%)	14 (45.2%)	47 (52.2%)
Non-GCB	26 (44.1%)	17 (54.8%)	43 (47.8%)
Missing	4	18	22
Days from diagnosis to randomization			
*N*	63	49	112
Mean (SD)	16.9 (9.38)	25.5 (14.72)	20.7 (12.71)
Median	14.0	26.0	18.0
Range	4.0, 42.0	1.0, 67.0	1.0, 67.0

*IPI* International Prognostic Index, *CNS-IPI* central nervous system-IPI, *COO* cell of origin, *GCB* germinal center B-cell

Patients characteristics included the following: 68 (60.7%) patients; Ann Arbor advanced stage III–IV in 98 (87.5%) cases; B symptoms in 39 (41.1%) cases. According to IPI, patients were stratified as low/intermediate-low risk (0–2) in 49 (43.8%) cases and intermediate-high/high risk (3–5) in 63 (56.3%) cases. According to central nervous system (CNS)-IPI, patients were stratified as low risk CNS-IPI 0–1 in seven (6.3%), intermediate risk CNS-IPI 2–3 in 82 (73.2%) and high risk CNS IPI 4–6 in 23 (20.6%) patients. Excluding 22 (19.6%) patients that were not evaluable for COO IHC testing, GCB vs non-GCB was observed in 47 (42.0%) vs 43 (38.4%) cases respectively.

### Long-term follow-up outcome

#### MC078E trial and REAL07 trial

At a median FU of 5.1 years (y), 5y-PFS was 59% (95% CI, 48–73%) vs 69% (95% CI, 57–85%) (*p* = 0.09), 5y-TTP was 68% (95% CI, 57–81%) vs 72% (95% CI, 60–88%) (*p* = 0.24) and 5y-OS was 74% (95% CI, 63–865) vs 77% (95% CI, 64–92%) (*p* = 0.28). Since differences in the long-term outcomes between the two trials were observed to be not statistically significant, a combined analysis of the two cohorts was done.

### Whole cohort

**Fig. 1:**
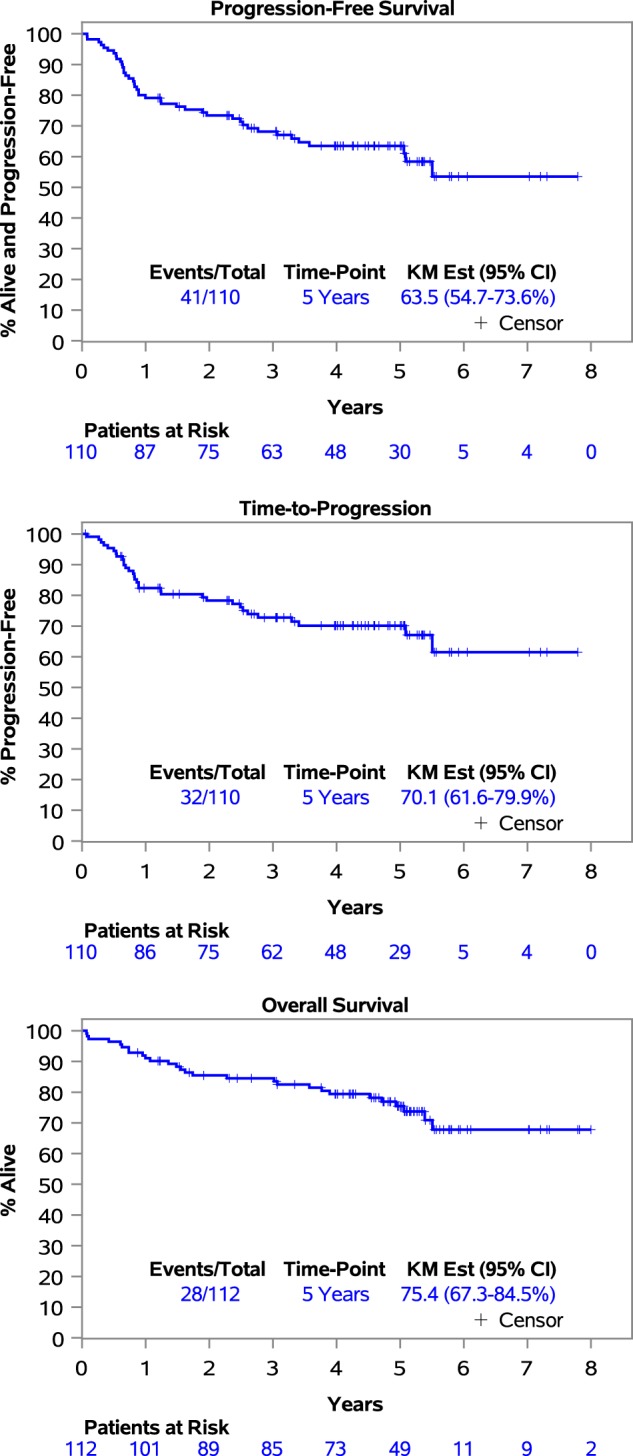
**Kaplan–Meier curves of progression-free survival, time to progression, overall survival of the whole cohort**

At a median follow-up of 5.1 years (y), for the whole cohort, 5y-PFS was 63.5% (95% CI, 54.7–73.6%), 5y-TTP was 70.1% (95% CI, 61.6–79.9%) and 5y-OS was 75.4% (95% CI, 67.3–84.5%) (Fig. 1). A total of 32 relapses were observed, with only 2 cases of CNS relapse. In the two patients who experienced CNS-relapse, the time from randomization to CNS-relapse was 287 and 183 days, the CNS-IPI was 4 and 3, COO was GCB and non-GCB, respectively; no intrathecal CNS phrophylaxis was administered in these two patients. Late relapse occurring beyond 3 years was observed in four cases (three cases with GCB phenotype and one case with missing COO data).

Twenty-five patients died with the following causes: lymphoma in 15 (60%) patients, late toxicities in one (4%), second tumors in three (12%), and other causes not related to hematological disease or treatment in six (24%) patients (one due to violent cause, one due to diabetes mellitus type 2 complications, three due to acute cardiorespiratory arrest and one due to bacteriemia, all non-related to lymphoma or treatment according to investigators).

**Fig. 2:**
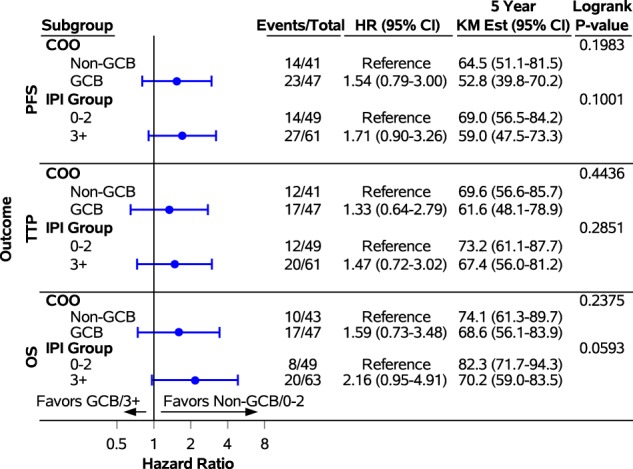
Forest plot of progression-free survival, time to progression and overall survival in a subgroup analysis based on International Prognostic Index and cell of origin. PFS progression-free survival, TTP time to progression, OS overall survival, IPI International Prognostic Index, COO cell of origin, GCB germinal center B-cell

**Fig. 3:**
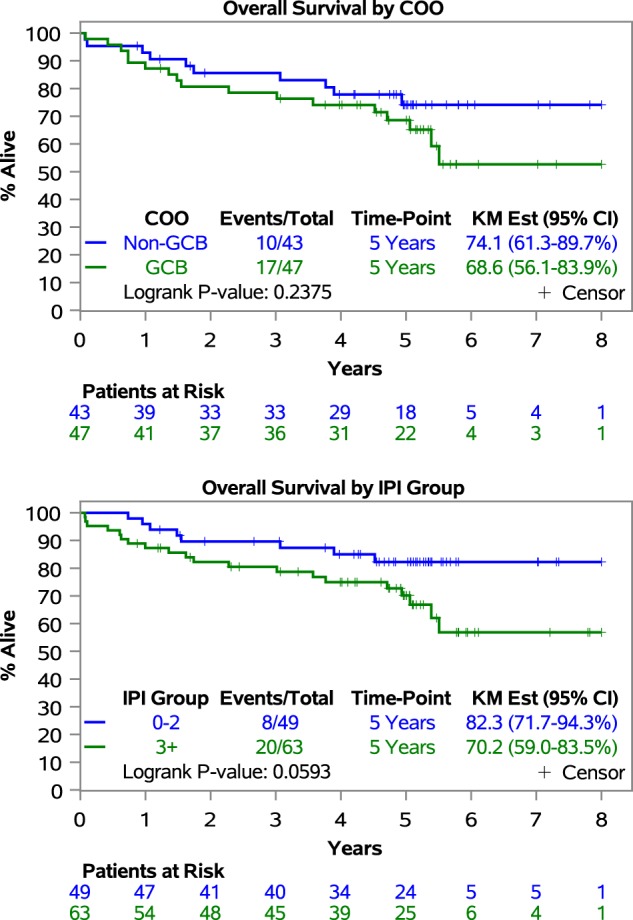
Kaplan–Meier curves of overall survival in a subgroup analysis based on International Prognostic Index and cell of origin. IPI International Prognostic Index, COO cell of origin, GCB germinal center B-cell

Outcome results in a subgroup analysis stratifying patients according to COO were: 5y-PFS was 52.8% (95% CI, 39.8–70.2%) vs 64.5% (95% CI, 51.1–81.5%) (*p* = 0.198), 5y-TTP was 61.6% (95% CI, 48.1–78.9%) vs 69.6% (95% CI, 56.6–85.7%) (*p* = 0.444) and 5y-OS was 68.6% (95% CI, 56.1–83.9%) vs 74.1% (95% CI, 61.3–89.7%) (*p* = 0.238) in GCB vs non-GCB respectively (Fig. 2). Outcome results in a subgroup analysis stratifying patients according to IPI 0–2 vs 3–5 were: 5y-PFS was 69.0% (95% CI, 56.5–84.2%) vs 59.0% (95% CI, 47.5–73.3%) (*p* = 0.100), 5y-TTP was 73.2% (95% CI, 61.1–87.7%) vs 67.4% (95% CI, 56.0–81.2%) (*p* = 0.285) and 5y-OS was 82.3% (95% CI, 71.7–94.3%) vs 70.2% (95% CI, 59.0–83.5%) (*p* = 0.059) (Figs. 2 and 3).

### Late toxicities and second tumors

Only one toxic death has been recorded in the follow-up period: a grade 5 sepsis occurred 6 months after the treatment completion in patient that was not neutropenic. Three patients experienced a severe grade 4 late toxicity (all grade 4 persistent neutropenia, subsequently resolved). Other milder grade ≤ 3 toxicities were: infections (in four cases, one grade 3 Gram negative bacteriemia and three cases of grade 1–2: viral infections in one case, Gram positive infections in two cases), thrombosis (one case grade 2) and persistent neuropathy (three cases, all grade 1–2). Two cases of cardiovascular disease grade 3 were reported.

Second malignancies were observed in seven patients (6.3%): one (0.9%) case of therapy-related secondary acute myeloid leukemia; six cases of other not therapy-related second tumors (two (1.8%) cases of second lymphoma (one T-cell and one mucosa-associated lymphoid tissue (MALT) lymphoma), one metastatic adenocarcinoma of unknown origin, one prostatic cancer, one rectal adenocarcinoma, and one non-melanotic tumor of the skin). Three patients died due to the occurence of the second tumor. The median time from the end of treatment to the second neoplasia onset is 16.4 months (range: 5.7–53.3 months).

## Discussion

This long-term follow-up analysis demonstrates that in patients with de novo DLBCL the R2-CHOP21 regimen maintained high efficacy over time, with high rates of PFS, TTP, and OS and with a good long-term safety profile.

At a median follow-up of 5.1 years (y), for the whole cohort, 5y-PFS was 63.5% (95% CI, 54.7–73.6%), 5y-TTP was 70.1% (95% CI, 61.6–79.9%) and 5y-OS was 75.4% (95% CI, 67.3–84.5%) (Fig. 1).

These results find a favorable comparison with historical data on patients treated with standard R-CHOP. Nowakowski et al.^22^, in a control cohort of DLBCL patients treated with R-CHOP alone, obtained from Mayo Clinic Database, showed a 2y-PFS of 52%; while in our present cohort treated with R-CHOP in association to lenalidomide 5y-PFS in the whole DLBCL cohort was 63.5%. These results are promising on a role of the addiction of lenalidomide to standard front-line chemoimmunotherapy in DLBCL setting.

Two randomized studies^25,26^ have completed accrual and are expected to be reported in the near future. E1412 is a randomized phase 2 study, which enrolled patients with newly diagnosed DLBCL regardless of COO and is powered to analyse COO results separately. Robust trial is a phase 3 registrational study that enrolled patients with ABC DLBCL only. These long-term efficacy and safety data in the present analysis will aid in the interpretation of the early results of these randomized trials.

In terms of efficacy, the current analysis demonstrates continuous benefit of R2CHOP with only a few relapses beyond year 2. Interestingly, late relapses appeared to occur in the GCB subtype.

In a subgroup analysis by the clinical prognostic index IPI, we can observe that IPI remained almost significantly predictive of survival (with patients with IPI score > 3 showing inferior OS, *p* = 0.059).

However, if we compared the outcome results of patients with intermediate-high/high IPI risk treated with R2-CHOP in our cohort, to historical data of IPI > 3 DLBCL treated with R-CHOP standard, we observed a 3y-OS of 80.5% vs 65.1% (IPI 3) and 59.0% (IPI 4–5) in patients treated with R2-CHOP vs historical R-CHOP treated control cohort, respectively^28^. These data suggested a role of association of lenalidomide to standard R-CHOP in improving the prognosis also of intermediate-high/high risk patients.

Both MC078E and REAL07 trials performed an exploratory analysis of patients treated with the combination regimen R2-CHOP21 in GCB vs non-GCB phenotype, suggesting that the addition of lenalidomide could mitigate the negative prognostic impact of the non-GCB subtype^22,24^. In the MC078E study^22^, a cohort of consecutive DLBCL patients, stage II–IV, from the Mayo Clinic Lymphoma Database treated with standard R-CHOP21 was retrospectively analyzed as a control. The outcome of the non-GCB phenotype was inferior to that of GCB in DLBCL patients treated with R-CHOP21 alone (2y-PFS in non-GCB vs GCB subtype of 28% vs 64%, respectively, *p* = 0.001), while there was no difference in outcome between the non-GCB vs GCB subtype in patients treated with R2-CHOP21 combination regimen. The current analysis demonstrates a durable benefit of R2-CHOP in non-GCB DLBCL, while results on GCB DLBCL showed to be superimposable to what obtained with standard R-CHOP^22^. This long-term response seems to translate into an apparent survival benefit in non-GCB DLBCL when compared to historical controls (2y-OS in R-CHOP treated cohort was 46% vs 5y-OS of 74.1% reported in our cohort of non-GCB DLBCL patients treated with R2-CHOP). Moreover, R2-CHOP21 was effective in patients with a high-intermediate/high IPI score and an older age (44.6% and 4.5% were over 70 and 80 years old, respectively).

A more accurate definition of COO, if compared with IHC, could be provided by GEP analysis such as the Nanostring® platform. In order to better evaluate the role of the association of lenalidomide to standard chemoimmunotherapy in different COO phenotypes, Nanostring® analysis in these patients is ongoing. Other recent trials to improve front-line outcomes of DLBCL patients investigated new regimens in both GCB and non-GCB/ABC subtypes. In the recent published GOYA trial^13^, which included more patients with a low-intermediate IPI risk score compared to the population of our study, a subgroup analysis according to COO determined by GEP was performed. A 3y-PFS of 75%, 59%, and 63% for the GCB, ABC, and unclassified phenotype, respectively, was reported with no difference between the rituximab and obinotuzumab arms. When compared to the 3y-PFS of patients treated with R2-CHOP in our analysis, the combination of lenalidomide may influence outcomes in non-GCB subtype DLBCL (3y-PFS of 61.2% and 67.4% in GCB and non-GCB respectively). The role of the combination of lenalidomide with standard R-CHOP21 in other subgroups of DLBCL with worse prognosis, such as MYC/BCL2 double expressors or double hit lymphomas remains to be defined, and further studies are ongoing. Future directions on the optimal use of lenalidomide toward a tailored therapy in DLBCL patients could come from the most recent advances in genetic and molecular knowledge of DLBCL^29^.

Despite a large number of intermediate/high CNS-IPI patients, CNS recurrences were less than expected, suggesting a role of lenalidomide combination in decreasing the risk of CNS involvement. There is evidence that small molecules, such as lenalidomide, could cross the blood–brain barrier. Lenalidomide also has been demonstrated to have single-agent activity in primary CNS lymphomas. In addition, non-GCB/ABC DLBCL are more likely to involve the CNS, and improved outcomes in this subgroup of patients could contribute to a reduced CNS-relapse rate^30^.

The addition of a new drug to standard chemoimmunotherapy raises concerns of increased toxicities, especially in older patients. This long-term follow-up analysis demonstrates that no new worrisome safety signals were observed in patients treated with an R2-CHOP21 combination regimen. In 112 patients, only one toxic death has been recorded in the follow-up period, a grade 5 sepsis that occurred 6 months after the completition of treatment. The other grade 4 late toxicities were grade 4 persistent neutropenia and all subsequently resolved. Among the four cases of infections reported, only two cases occurred in neutropenic patients (the case of grade 3 Gram negative bacteriemia and one grade 1–2 infection), and both resolved. The only patient death secondary to sepsis was not related to neutropenia at the time of the event. In keeping with earlier published results which suggested that the incidence of neuropathy was low^21–24^ with R2CHOP21, long-term analysis shows only three patients reported a persistent neuropathy, all mild/grade 1–2. Two cases of cardiovascular disease grade 3 were observed, but it must be noted that the median age of patients was high and cardiotoxicity could be attributed mainly to antracycline-based therapy.

The incidence of second malignancies was comparable rates in patients treated with RCHOP^31^. Moser et al., reported, for patients treated with CHOP or CHOP like therapy, a cumulative incidence at 15 years of therapy-related secondary acute leukemia/myelodysplastic syndrome and other tumors of 3% and 11% respectively^30^. In our cohort, the cumulative incidence at 5 years was 0.9% and 5.4% respectively. Indeed, considering the median age of patients, prostatic cancer and tumor of the skin are quite common in this population.

In conclusion, our long-term follow-up combined analysis from two phase II trials shows that R2-CHOP21 efficacy was maintained over time with a high rate of PFS, TTP, and OS, considering high risk feature patients that were included. The addition of lenalidomide to RCHOP appears to mitigate the negative prognostic impact of non-GCB phenotype. The incidence of therapy related secondary malignancies was low, and no new worrisome long-term safety concerns were reported. Phase III randomized trials have recently concluded, with early results expected in the near future. These long-term efficacy and safety data will aid in the interpretation of the results.
